# A Randomized, Placebo‐Controlled, Blinded Clinical Trial Evaluating PCSO‐524 as an Adjunctive Therapy for Noninfectious Pododermatitis in Rabbits

**DOI:** 10.1155/vmi/4177859

**Published:** 2025-12-14

**Authors:** Walasinee Sakcamduang, Nijanan Siriarchawattana, Phattanan Korjaranjit, Vitthanan Natepoo, Nawarat Suriyakhun, Chaowaphan Yinharnmingmongkol, Mookmanee Taechikantaphat

**Affiliations:** ^1^ Department of Clinical Sciences and Public Health, Faculty of Veterinary Science, Mahidol University, Nakhon Pathom, 73170, Thailand, mahidol.ac.th; ^2^ Faculty of Veterinary Science, Mahidol University, Nakhon Pathom, 73170, Thailand, mahidol.ac.th; ^3^ Prasu Arthorn Veterinary Teaching Hospital, Faculty of Veterinary Science, Mahidol University, Nakhon Pathom, 73170, Thailand, mahidol.ac.th; ^4^ Animal Space Pet Hospital, Thawi Watthana, Bangkok, 10170, Thailand

**Keywords:** inflammation, omega-3 fatty acids, PCSO-524, pododermatitis, rabbit

## Abstract

This study evaluated the efficacy and safety of PCSO‐524, a natural supplement containing omega‐3 fatty acids, as an adjunctive therapy for noninfectious pododermatitis (Grades 1‐2) in rabbits. In a randomized, placebo‐controlled, blinded trial, 23 rabbits with noninfectious pododermatitis received either PCSO‐524 or a placebo along with standard treatments for 56 days. PCSO‐524 supplementation led to a statistically significant reduction in the mean lesion size of both hind limbs within the PCSO group compared to baseline (Day 0: 98.55 ± 52.75 mm^2^, Day 56: 53.18 ± 21.54 mm^2^; *p* = 0.002), while the placebo group did not show significant changes (Day 0: 93.98 ± 43.88 mm^2^, Day 56: 75.61 ± 115.68 mm^2^; *p* = 0.58). Both groups exhibited alterations in white blood cell populations (increased monocytes, decreased eosinophils) within normal ranges, and a consistently elevated heterophil‐to‐lymphocyte ratio (HLR; > 1:1). No adverse events were associated with PCSO‐524. This study provides initial evidence for the safety and potential efficacy of a natural supplement containing omega‐3 fatty acids as an adjunctive therapy for noninfectious pododermatitis in rabbits.

## 1. Introduction

Pododermatitis, a common inflammatory skin condition in rabbits, birds, and rodents, significantly impacts animal health and welfare [1]. Rabbits are particularly susceptible due to their thin plantar skin and lack of footpads, predisposing them to pressure sores and subsequent complications [2, 3]. Several factors contribute to pododermatitis in rabbits. Predisposing factors include breed (e.g., short‐coated breeds like Rex having less protective fur) and body size (e.g., large breeds like Flemish Giant). Anatomical abnormalities leading to abnormal weight distribution also increase risk. Environmental factors include unsuitable flooring (e.g., wire cages), lack of mobility due to restricted space, and unhygienic housing contaminated with urine or feces [2, 4–6].

The pathogenesis of pododermatitis typically begins with pressure‐induced ischemia and necrosis of the plantar skin. Reduced blood perfusion leads to free radical damage, thrombosis, and progressive ulceration. Untreated lesions or improper management can result in secondary bacterial infections and severe inflammation [1, 5]. The severity of pododermatitis is often classified using a grading system, with early signs including hair loss and calluses, and severe cases progressing to ulcers, abscesses, and even osteomyelitis [5, 7, 8]. Current pododermatitis treatments in rabbits focus on addressing underlying causes, managing pressure sores, controlling infection, and reducing pain. Surgery is rarely recommended due to insufficient skin for flaps [1, 2, 5].

Despite existing treatments, pododermatitis management remains challenging. PCSO‐524, a natural supplement derived from New Zealand green mussels (*Perna canaliculus*), offers potential as an alternative or adjunctive therapy. Its anti‐inflammatory properties stem from omega‐3 polyunsaturated fatty acids (PUFAs) that act on multiple pathways. PUFAs can inhibit cyclooxygenase (COX) enzymes, thus interrupting arachidonic acid metabolism and reducing inflammation. Furthermore, they compete with inflammatory precursors and modulate leukotriene and interleukin synthesis [9–13]. While promising in other species, the efficacy of PCSO‐524 in treating rabbit pododermatitis remains unexplored.

This study investigated the potential of PCSO‐524 as a supplementary treatment for noninfectious pododermatitis (Grades 1‐2) in rabbits. We hypothesized that rabbits receiving PCSO‐524 in addition to standard care would exhibit a greater reduction in pododermatitis severity compared to those receiving a placebo. The study aimed to evaluate changes in lesion size and hematological parameters over a 56‐day period.

## 2. Materials and Methods

### 2.1. Animals

This study enrolled 30 client‐owned rabbits presenting with noninfectious pododermatitis (Grades 1‐2) between June 2017 and December 2017. Rabbits with other local or systemic diseases that may affect pododermatitis were excluded from the study. These included, but were not limited to, arthritis, spondylosis, dental disease, otitis, *E. cuniculi* infection, obesity, rhinitis, and limb amputation. Participation was with informed owner consent, and the study was approved by the Mahidol University‐Institute Animal Care and Use Committee of the Faculty of Veterinary Science (No. MUVS‐2017‐06‐16). An experienced exotic animal veterinarian (N.S.) confirmed diagnoses using the Pet Rabbit Pododermatitis Scoring System (PRPSS) [8]. Pododermatitis severity was graded according to this system, and we collected comprehensive data for each rabbit, including signalment, wellness care, housing conditions, and detailed physical examination findings.

Prior to treatment initiation (Day 0), each rabbit underwent a detailed initial physical examination and assessment to establish baseline pododermatitis severity. On Days 0, 28, and 56 postenrolment, blood collections were performed, and follow‐up examinations were conducted. These follow‐ups included a detailed physical examination, evaluation of clinical response, and reassessment of pododermatitis severity. For blood collection, 1.5–2 mL was drawn from the lateral saphenous vein using a 24‐G needle and a 3‐mL syringe. The blood was divided into two parts: an EDTA tube (0.5 mL) for complete blood count (CBC) using an Animal Blood Counter ABX Micros ESV60 (HORIBA ABX Diagnostic (Thailand) Ltd., Bangkok, Thailand), following manufacturer‐recommended settings for rabbit hematology, and a plain tube (1–1.5 mL) centrifuged to separate serum for subsequent analysis of BUN, creatinine, total protein, and albumin using a Sapphire 400 Auto‐Chemistry Analyzer (D.A.P. Siam Group Ltd., Bangkok, Thailand) [14].

Initially, 30 rabbits were recruited. Due to loss to follow‐up, seven rabbits were excluded from the final analysis. The randomization method and comparability of groups after the loss to follow‐up will be addressed in the following section.

### 2.2. Study Groups and Interventions

Rabbits enrolled in the study were randomly assigned to one of two groups using stratified randomization based on breed distribution. Within each breed, rabbits were randomly allocated to either the PCSO‐524 group or the placebo group using a random number generator. This stratification ensured a balanced distribution of breeds across the two treatment groups. To maintain comparability after the loss of seven rabbits, we analyzed the baseline characteristics (age, sex, breed, and lesion size) of the excluded rabbits and found no significant differences between those who completed the study and those who were lost to follow‐up.

- PCSO‐524 group: Rabbits received standard pododermatitis treatment plus daily PCSO‐524 supplementation. Each 50‐mg capsule of PCSO‐524 contains 35% (±5%) DHA and EPA by weight. The composition of a 50‐mg capsule is as follows: *Perna canaliculus* oil (50 mg), vitamin E (0.225 mg), and other ingredients, including olive oil, gelatin, and glycerin [15].
- Placebo group: Rabbits received standard pododermatitis treatment plus a visually identical placebo (50 mg sunflower oil and other ingredients, including gelatin and glycerin) [15].

To maintain study integrity, a double‐blind approach was employed. Neither investigators nor owners were aware of the specific intervention (PCSO‐524 or placebo) administered to each rabbit. Treatments were labeled only as “Med A” or “Med B.”

In cases of refusal to voluntarily ingest the capsule, the capsule contents were aspirated using a sterile 1‐mL syringe fitted with an 18‐gauge needle. The needle was removed before gently administering the aspirated fluid orally to the rabbit.

Standard pododermatitis treatment consisted of topical application of mupirocin cream and the provision of a thick layer of clean bedding. Housing conditions were modified to eliminate direct contact between the rabbits′ feet and the wire cage flooring.

Rabbits in both groups received a 56‐day treatment period beginning on the day of enrollment (Day 0, Visit 1). Follow‐up visits occurred on Days 28 and 56 for clinical response evaluation and reassessment of pododermatitis severity. To maintain study integrity, researchers remained blinded to treatment assignments (PCSO‐524 vs. placebo) until after data analysis was completed.

### 2.3. Assessment of Pododermatitis Lesions

Lesion severity was assessed using the PRPSS [5], providing a standardized approach for comparison before and after treatment. This system defines Grades 1 and 2 as follows: Grade 1 pododermatitis presents as a small circular area with minimal alopecia, hyperemia, and/or hyperkeratosis of the skin, but no evidence of infection or bleeding of underlying tissues. Grade 2 pododermatitis presents as a circumscribed area with alopecia, erythema, and scaling with no signs of infection [5, 8]. Standardized photographs of the left and right hocks of each rabbit were captured at each visit using a digital camera (Canon G12, Japan). To ensure consistent image analysis, a ruler was included in each photograph as a reference for scale (Figure 1). Three independent, blinded investigators scored these images. To quantify lesion extent, the area of each lesion was measured using ImageJ software. Each investigator measured every image three times, with the median value used as the representative area. The final lesion area was determined by calculating the median score among the three investigators.

Figure 1Visual grading of pododermatitis lesions. (a) Grade 1 pododermatitis: a small circular area with minimal alopecia, hyperemia, and/or hyperkeratosis of the skin, but no evidence of infection or bleeding of underlying tissues. (b) Grade 2 pododermatitis: a circumscribed area with alopecia, erythema, and scaling with no signs of infection.

**Figure:**
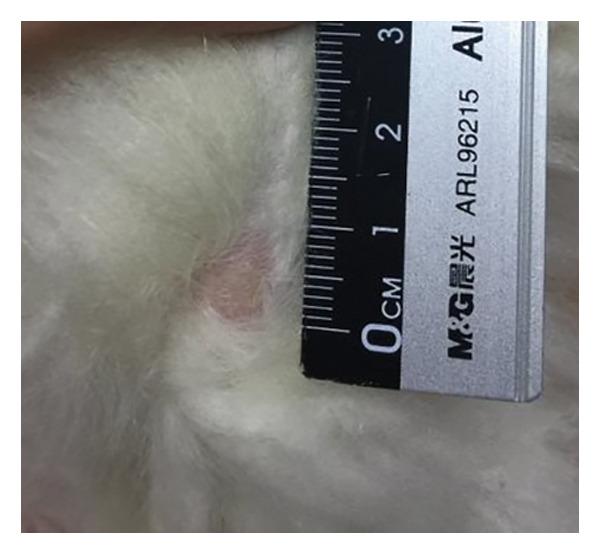
(a)

**Figure:**
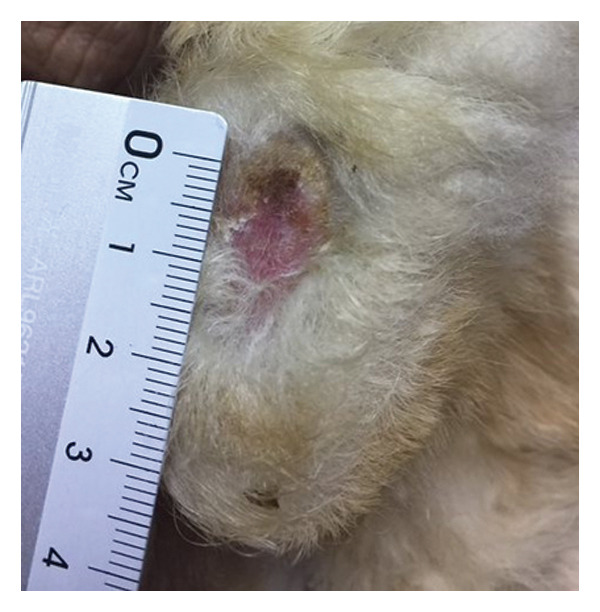
(b)

### 2.4. Statistical Analysis

Data analysis was performed using IBM SPSS Statistics (Version 27) for Windows (Chicago, IL, USA). A significance level of *p* < 0.05 was used for all statistical tests. Baseline categorical variables were compared between groups using the chi‐square test. The Shapiro–Wilk test was used to assess the normality of distribution for numerical parameters within each treatment group on each examination day. To compare numerical variables between groups at each examination time point, the independent *T*‐test or Welch’s *t*‐test for equal or unequal variances was employed appropriately. For within‐group comparisons of numerical variables (Day 0 vs. Day 28, Day 0 vs. Day 56, and Day 28 vs. Day 56), the one‐way repeated‐measures ANOVA was used, followed by the paired *T*‐test for post hoc pairwise comparisons.

To assess the power of the analysis to detect a clinically meaningful difference within subjects, we calculated the sample size needed to detect a 40% reduction in lesion area in the last visit from baseline with 80% power and a significance level of 0.05. Based on the observed standard deviation of lesion size in the pilot study, a sample size of 10 rabbits per group was determined to be sufficient.

## 3. Results

### 3.1. Study Population and Breed Distribution

Twenty‐three rabbits completed the 56‐day study protocol, representing five breeds: mixed breeds (*n* = 10), Holland Lop (*n* = 8), Mini Rex (*n* = 3), Netherland Dwarf (*n* = 1), and Woody Toy (*n* = 1). They were divided into a PCSO‐524 group (*n* = 13) and a placebo group (*n* = 10). The PCSO‐524 group consisted of 5 mixed breeds, 4 Holland Lop, 2 mini Rex, and one each of Netherland Dwarf rabbits and Woody Toy. The placebo group included 5 mixed breeds, 4 Holland Lop, and 1 mini Rex. No statistically significant difference in breed distribution was observed between the groups (*p* = 0.74).

Baseline characteristics, including age, sex, and body weight, were also compared (Table 1). The mean age of rabbits in the PCSO‐524 group was 2.92 ± 0.76 years, and the mean age of rabbits in the placebo group was 3.44 ± 1.24 years (*p* = 0.23). Sex distribution in the PCSO‐524 group was 10 males and 3 females, compared to 5 males and 5 females in the placebo group (*p* = 0.18). The average body weight in the PCSO‐524 group was 2.2 ± 0.8 kg, and that in the placebo group was 1.9 ± 0.3 kg (*p* = 0.54). No statistically significant differences were found between groups with respect to age, sex, or body weight.

**Table 1:** Statistical comparisons of variables across visits and between treatment groups.

Variables	Visit	PCSO‐524 (*n* = 13)	*p* value within PCSO‐524 group	Placebo (*n* = 10)	*p* value within placebo group	*p* value between PCSO‐524 vs. placebo group
Age	1	2.92 ± 0.76	—	3.44 ± 1.24	—	0.23
Sex (male/female)	1	10/3	—	5/5		0.18
Body weight (kg)	1	2.2 ± 0.8		1.9 ± 0.3		0.54

Lesion size: right (mm^2^)	1	47.62 ± 30.63^a,b^	0.001	53.34 ± 20.45	0.32	0.62
	2	34.51 ± 17.55^a,c^		52.17 ± 57.77		0.31
	3	24.78 ± 13.53^b,c^		41.12 ± 69.77		0.42

Lesion size: left (mm^2^)	1	50.93 ± 32.85^d,e^	0.006	42.38 ± 24.38	0.42	0.50
	2	32.92 ± 15.17^d^		26.67 ± 12.93		0.31
	3	29.17 ± 11.10^e^		34.49 ± 46.43		0.69

Lesion size: right and left (mm^2^)	1	98.55 ± 52.75^f,g^	< 0.001	93.98 ± 43.88	0.58	0.83
	2	68.01 ± 25.53^f,h^		78.84 ± 67.90		0.60
	3	53.18 ± 21.54^g,h^		75.61 ± 115.68		0.50

White blood cell count (10^3^/μL)	1	7.14 ± 2.13	0.84	7.52 ± 1.90	0.08	0.66
	2	7.36 ± 1.81		6.53 ± 1.54		0.26
	3	6.98 ± 2.20		6.52 ± 1.94		0.60

Monocytes (10^3^/μL)	1	0.03 ± 0.07^i,j^	0.007	0.03 ± 0.09^k^	0.04	0.91
	2	0.12 ± 0.08^i^		0.13 ± 0.06^k^		0.92
	3	0.15 ± 0.10^j^		0.10 ± 0.05		0.16

Heterophils (10^3^/μL)	1	3.51 ± 1.82	0.50	4.39 ± 1.33	0.11	0.22
	2	4.04 ± 1.39		3.49 ± 1.05		0.30
	3	3.50 ± 1.56		3.66 ± 1.38		0.80

Lymphocytes (10^3^/μL)	1	3.33 ± 1.40	0.55	2.49 ± 1.10	0.90	0.13
	2	3.03 ± 1.65		2.61 ± 0.95		0.48
	3	3.14 ± 1.34		2.52 ± 0.82		0.22

Eosinophils (10^3^/μL)	1	0.17 ± 0.09^l,m^	< 0.001	0.23 ± 0.20^n,o^	0.003	0.36
	2	0.02 ± 0.04^l^		0.02 ± 0.04^n^		0.78
	3	0.06 ± 0.07^m^		0.05 ± 0.04^o^		0.65

Basosinophils (10^3^/μL)	1	0.09 ± 0.07	0.50	0.10 ± 0.12	0.10	0.87
	2	0.14 ± 0.15		0.22 ± 0.17		0.22
	3	0.13 ± 0.16		0.10 ± 0.12		0.61

HLR	1	1.22 ± 0.73	0.06	1.93 ± 0.76	0.61	0.03
	2	1.67 ± 0.85		1.64 ± 1.23		0.95
	3	1.24 ± 0.58		1.53 ± 0.60		0.25

Erythrocytes (10^6^/μL)	1	5.60 ± 0.60	0.64	5.49 ± 0.91	0.93	0.72
	2	5.60 ± 0.60		5.51 ± 0.68		0.75
	3	5.68 ± 0.77		5.45 ± 0.51		0.43

Hemoglobin (g/dL)	1	12.3 ± 1.2	0.90	11.9 ± 1.5	0.80	0.50
	2	12.3 ± 1.3		12.1 ± 1.1		0.65
	3	12.4 ± 1.4		11.9 ± 0.8		0.31

PCV (%)	1	37.9 ± 3.4	0.60	37.2 ± 5.0	0.93	0.74
	2	38.5 ± 3.7		37.0 ± 2.5		0.28
	3	38.3 ± 4.3		37.0 ± 2.8		0.37

MCV (fL)	1	67.7 ± 3.4	0.21	67.4 ± 3.9	0.23	0.83
	2	68.8 ± 3.3		66.8 ± 4.5		0.22
	3	67.7 ± 3.7		67.3 ± 3.3		0.80

MCH (pg)	1	22.0 ± 1.0	0.54	21.5 ± 1.1	0.83	0.32
	2	22.0 ± 0.9		21.6 ± 1.1		0.40
	3	21.9 ± 0.8		21.7 ± 1.1		0.62

MCHC (g/dL)	1	32.5 ± 0.8	0.23	32.0 ± 1.3	0.64	0.28
	2	32.0 ± 0.8		32.5 ± 1.4		0.31
	3	32.3 ± 1.0		32.2 ± 0.8		0.77

Platelets (10^3^/μL)	1	404.2 ± 82.8	0.95	423.3 ± 87.0	0.12	0.28
	2	404.1 ± 77.0		418.8 ± 83.6		0.67
	3	398.0 ± 98.7		381.4 ± 99.7		0.70

RDW (%)	1	12.4 ± 0.4	0.49	12.8 ± 0.9	0.61	0.16
	2	12.5 ± 0.8		13.1 ± 1.7		0.25
	3	12.3 ± 0.4		13.0 ± 1.2		0.07

Plasma protein (g/dL)	1	8.0 ± 0.3	0.82	7.8 ± 0.4	0.34	0.36
	2	8.0 ± 0.5		8.0 ± 0.5		0.89
	3	8.0 ± 0.4		7.8 ± 0.5		0.45

Fibrinogen (g/dL)	1	0.25 ± 0.12	0.54	0.26 ± 0.13	0.17	0.79
	2	0.29 ± 0.16		0.26 ± 0.10		0.57
	3	0.25 ± 0.12		0.18 ± 0.12		0.19

*Note:* HLR: heterophil‐to‐lymphocyte ratio; RDW: red cell distribution width.

Abbreviations: MCH, mean corpuscular hemoglobin; MCHC, mean corpuscular hemoglobin concentration; MCV, mean corpuscular volume; PCV, packed cell volume.

^a^A significant decrease from Visit 1 to Visit 2 within the PCSO group (*p* = 0.03).

^b^A significant decrease from Visit 1 to Visit 3 within the PCSO group (*p* = 0.004).

^c^A significant decrease from Visit 2 to Visit 3 within the PCSO group (*p* = 0.02).

^d^A significant decrease from Visit 1 to Visit 2 within the PCSO group (*p* = 0.02).

^e^A significant decrease from Visit 1 to Visit 3 within the PCSO group (*p* = 0.02).

^f^A significant decrease from Visit 1 to Visit 2 within the PCSO group (*p* = 0.007).

^g^A significant decrease from Visit 1 to Visit 3 within the PCSO group (*p* = 0.002).

^h^A significant decrease from Visit 2 to Visit 3 within the PCSO group (*p* = 0.013).

^i^A significant increase from Visit 1 to Visit 2 within the PCSO group (*p* = 0.02).

^j^A significant increase from Visit 1 to Visit 3 within the PCSO group (*p* = 0.01).

^k^A significant increase from Visit 1 to Visit 2 within the placebo group (*p* = 0.04).

^l^A significant decrease from Visit 1 to Visit 2 within the PCSO group (*p* < 0.001).

^m^A significant decrease from Visit 1 to Visit 3 within the PCSO group (*p* = 0.01).

^n^A significant decrease from Visit 1 to Visit 2 within the placebo group (*p* = 0.02).

^o^A significant decrease from Visit 1 to Visit 3 within the placebo group (*p* = 0.02).

### 3.2. Visit Timing

Visit 2 occurred at an overall mean of 28.0 ± 2.5 days (range 24–35). The PCSO‐524 group mean was 28.2 ± 2.7 days (range 24–35), and the placebo group mean was 27.9 ± 2.2 days (range 24–33). No statistically significant difference in Visit 2 timing was observed between groups (*p* = 0.81).

Visit 3 occurred at an overall mean of 56.4 ± 4.8 days (range 49–63). The PCSO‐524 group mean was 56.7 ± 5.3 days (range 49–63), and the placebo group mean was 56.1 ± 4.3 days (range 49–63). There was no statistically significant difference in Visit 3 timing between groups (*p* = 0.78).

### 3.3. Changes in Lesion Size

The PCSO‐524 group exhibited significant reductions in lesion size over time for the left hind limb, right hind limb, and both limbs combined. Statistically significant decreases were observed between all successive visits for the PCSO‐524 group (see Figure 2). This pattern of lesion reduction was less pronounced in the placebo group, in which no significant decrease was observed across the three visits (Table 1, Figure 2). No statistically significant differences in lesion size were found between the PCSO‐524 and placebo groups at any individual visit (Table 1).

Figure 2Changes in lesion size of hind limbs in rabbits with pododermatitis. (a) Right hind limb, (b) left hind limb, and (c) both hind limbs combined. Plots show the mean ± SD at each visit for rabbits receiving PCSO‐524 (*n* = 13, filled squares) or placebo (*n* = 10, filled circles). Superscripts (a–h) indicate statistically significant differences between visits within the PCSO‐524 group.

**Figure:**
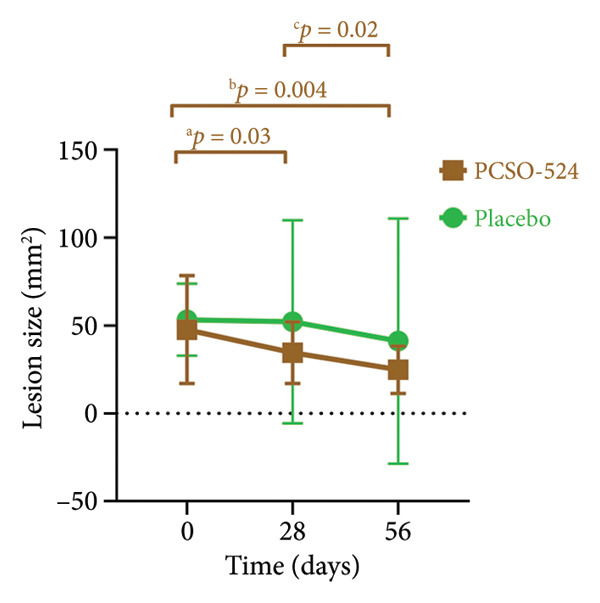
(a)

**Figure:**
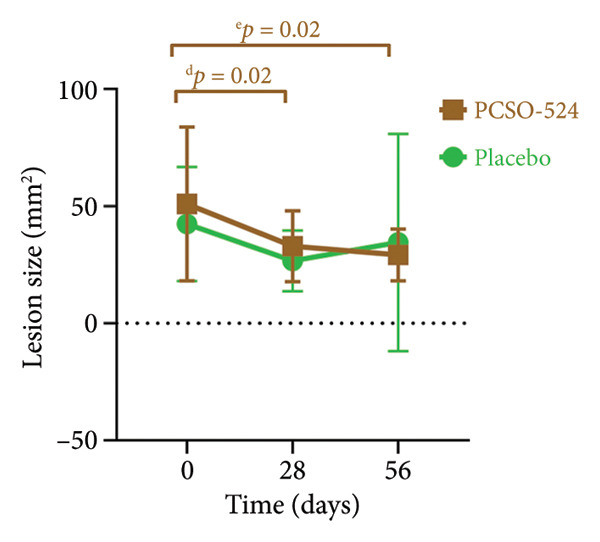
(b)

**Figure:**
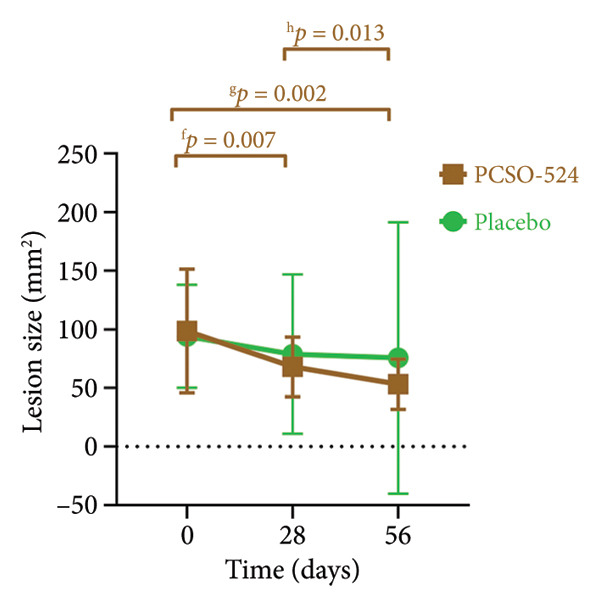
(c)

Figures 2(a), 2(b), and 2(c) visually illustrate the changes in lesion size for the right hind limb, left hind limb, and both limbs combined, respectively. The consistent downward trend in lesion size within the PCSO‐524 group, along with the corresponding statistical significance, is clearly depicted in Figures 2(a), 2(b), and 2(c).

### 3.4. Hematological Analysis

CBC analysis revealed that all hematological parameters, with the exception of the heterophil‐to‐lymphocyte ratio (HLR), remained within established normal reference ranges for healthy rabbits throughout the study for both the PCSO‐524 (*n* = 13) and placebo (*n* = 10) groups [16–18]. No statistically significant differences were observed between groups for individual cell counts at any visit, except that the HLR was higher in the first visit of the placebo group.

Both groups exhibited a significant increase in monocyte counts over the study period (see Table 1). Conversely, eosinophil counts showed a significant decrease throughout the study for both the PCSO‐524 and placebo groups (Table 1).

Interestingly, the HLR, while within the normal range, remained consistently higher than 1:1 in both groups throughout the study.

## 4. Discussions

This clinical trial evaluated whether adjunctive therapy with PCSO‐524, an omega‐3 fatty acid supplement, could improve clinical outcomes for rabbits with noninfectious pododermatitis (Grades 1‐2). The primary finding was a statistically significant reduction in lesion size within the PCSO‐524 group after 28 and 56 days of treatment, compared to baseline, while the placebo group showed no significant change. This suggests that PCSO‐524 may offer a beneficial adjunctive approach for managing pododermatitis in rabbits. This improvement is likely attributed to the anti‐inflammatory properties of omega‐3 fatty acids, which modulate inflammatory pathways associated with wound healing [9–11, 19–21]. Furthermore, the absence of adverse effects during the study supports the potential safety of PCSO‐524 in rabbits.

The observed reduction in lesion size aligns with the known anti‐inflammatory properties of omega‐3 fatty acids. These fatty acids have been shown to modulate inflammatory pathways by altering eicosanoid synthesis and influencing cytokine production [22, 23]. This anti‐inflammatory effect is crucial in the context of pododermatitis, a condition characterized by chronic inflammation and tissue damage [8]. Furthermore, omega‐3 fatty acids have been implicated in promoting wound healing, potentially contributing to the observed lesion size reduction [24, 25]. Our findings align with more recent studies that also highlight the role of omega‐3 fatty acids in modulating chronic inflammation in other animal models [13, 26, 27].

Interestingly, while overall hematological parameters remained within normal reference ranges, significant changes in specific white blood cell populations were observed. Both groups exhibited increased monocyte counts and decreased eosinophil counts throughout the study. Monocytes play a critical role in chronic inflammation and tissue remodeling. Their increase could reflect an ongoing effort to combat persistent inflammation associated with pododermatitis [17, 28]. Conversely, the decrease in eosinophils, which have complex roles in inflammatory responses, could be attributed to various factors, including the chronic nature of the condition [29].

Interestingly, while the HLR remained within normal reference ranges for both groups, it consistently exceeded a 1:1 ratio. Elevated HLR, recognized as a marker of systemic inflammation, is associated with various chronic inflammatory conditions in rabbits [14, 18, 29]. This elevated HLR is recognized as a marker of systemic inflammation, and its elevation has been observed in various chronic inflammatory conditions in both human and veterinary medicine, even when values are within the normal range [30–32]. This finding, coupled with the alterations in monocytes and eosinophils, strengthens the hypothesis that subclinical inflammation may contribute to pododermatitis persistence in rabbits.

Our hematological findings warrant further investigation. Larger studies are needed to confirm these trends and potentially establish links between specific hematological parameters and pododermatitis severity. Exploring the utility of HLR as a monitoring tool for disease progression or treatment response could prove valuable in managing this condition, as has been demonstrated in other diseases [14, 33]. Additionally, investigating therapies targeting monocyte activity or inflammation in general might offer novel avenues for improving pododermatitis management.

This study has several strengths, including its randomized, placebo‐controlled, and blinded design, which enhances the reliability of the findings. However, it also has limitations. The use of client‐owned rabbits may introduce variability in housing and husbandry practices. Furthermore, slight variations in follow‐up scheduling occurred due to the practicalities of a clinical setting with client‐owned animals; however, as visit timings were statistically similar between groups, this is unlikely to have biased the results. The relatively small sample size may limit the generalizability of the results. Furthermore, the 56‐day follow‐up period may not be sufficient to assess the long‐term effects of PCSO‐524.

Despite these limitations, our findings suggest that PCSO‐524 may be a safe and effective adjunctive therapy for pododermatitis in rabbits. Future research should focus on investigating the mechanisms of action of PCSO‐524, determining the optimal dosage, and evaluating its long‐term effects. Additionally, further studies are needed to explore the clinical significance of the observed hematological changes and their potential use in monitoring and managing pododermatitis.

## 5. Conclusion

This study demonstrates that PCSO‐524 supplementation, in conjunction with standard treatment protocols, may significantly improve pododermatitis lesion size in rabbits. These findings have important clinical implications for veterinarians managing this challenging condition.

## Ethics Statement

The study was approved by the Mahidol University–Institutional Animal Care and Use Committee of the Faculty of Veterinary Science (No. MUVS‐2017‐06‐16).

## Disclosure

The funder had no involvement in the study’s design, execution, data analysis, interpretation, or the decision to submit for publication.

## Conflicts of Interest

The authors declare no conflicts of interest.

## Funding

The authors gratefully acknowledge the funding support provided by Pharmalink International Ltd., Central, Hong Kong.

## Data Availability

The data that support the findings of this study are available from the corresponding author upon reasonable request.
