# Correction to “A Randomized, Placebo‐Controlled, Blinded Clinical Trial Evaluating PCSO‐524 as an Adjunctive Therapy for Noninfectious Pododermatitis in Rabbits”

**DOI:** 10.1155/vmi/9821075

**Published:** 2026-09-22

W. Sakcamduang, N. Siriarchawattana, P. Korjaranjit, et al., “A Randomized, Placebo‐Controlled, Blinded Clinical Trial Evaluating PCSO‐524 as an Adjunctive Therapy for Noninfectious Pododermatitis in Rabbits,” Veterinary Medicine International 2025 (2025): 4177859, https://doi.org/10.1155/vmi/4177859.

In the article titled “A Randomized, Placebo‐Controlled, Blinded Clinical Trial Evaluating PCSO‐524 as an Adjunctive Therapy for Noninfectious Pododermatitis in Rabbits,” there was an error in paragraph 1 of the “Materials and Methods” section. The text

“This study enrolled 30 client‐owned rabbits presenting with noninfectious pododermatitis (Grades 1‐2) between June 2017 and December 2017.” was incorrect.

This should have read:

“This study enrolled 30 client‐owned rabbits presenting with noninfectious pododermatitis (Grades 1‐2) between July 2017 and December 2017.”

We apologize for this error.

